# Fracture Resistance of Molars With Simulated Strip Perforation Repaired With Different Calcium Silicate-Based Cements

**DOI:** 10.7759/cureus.34462

**Published:** 2023-01-31

**Authors:** Alaa Kabtoleh, Ossama Aljabban, Yasser Alsayed Tolibah

**Affiliations:** 1 Endodontics, Damascus University, Damascus, SYR; 2 Pediatric Dentistry, Damascus University, Damascus, SYR

**Keywords:** fracture resistance, calcium-enriched mixture, bioceramic putty, mineral trioxide aggregate, strip perforation

## Abstract

Background and aims

A root perforation is a connection between the root canal system and the external supportive tissues. Strip perforation (SP), occurring within root canals in a strip, can worsen the prognosis of a treated tooth, reduce its mechanical resistance, and impair the tooth structure. One of the suggested methods to treat SP is to seal it with a bio-material such as calcium silicate cement. Therefore, this in vitro study aimed to assess the molar structure impairment due to SP, which requires studying the fracture resistance, and the ability of mineral trioxide aggregate (MTA), bioceramic, and calcium-enriched mixture (CEM) to repair this perforation.

Materials and methods

Seventy-five molars were instrumented to size #25 and taper 4%, irrigated with sodium hypochlorite and ethylenediaminetetraacetic acid (EDTA), dried, and then divided randomly into five groups (G1-G5): in G1, root canals were filled with gutta-percha and sealer (negative control sample), whereas the rest of the groups (G2-G5) had a manual simulated SP made with Gates Glidden drill at the mesial root of the extracted molar, and filled with gutta-percha and sealer up to their perforation area; in G2, SP was filled with gutta-percha and sealer (positive control sample); G3 used MTA to repair the SP; G4 used bioceramic putty; and G5 used CEM. Fracture resistance tests of the molars were conducted in the crown-apical direction using a universal testing machine. One-way ANOVA test and Bonferroni test were used to study the significance of the differences in the mean values of the tooth fracture resistance, where statistical significance was set at 0.05.

Results

The ANOVA test showed that there were statistically significant differences between the fracture resistance (in newtons) values among groups (p = 0.000). The Bonferroni test showed that G2 had a smaller fracture resistance mean than the other four study groups (656.53 N; p = 0.000), and that of G5 was smaller than G1, G3, and G4 (794.40 N, 1083.73 N, 1025.20 N, and 1034.20 N, respectively; p = 0.000 in each pairwise comparison).

Conclusion

SP reduced the fracture resistance of endodontically treated molars. SP restored using MTA and bioceramic putty was better than that treated with CEM and similar to molars without SP. Moreover, MTA and bioceramic putty enhanced the fracture resistance of endodontically treated teeth to levels similar to molars without SP.

## Introduction

Root canal treatment (RCT) is a therapeutic procedure with many potential complications that can occur at any stage of treatment. One such complication is perforation, which may occur during access cavity preparation, root canal instrumentation, post-space preparation, root canal preparation with internal or external resorption, or the removal of a considerable amount of dentin around separated instruments [1,2].

Root perforations can be classified according to their cause (iatrogenic or pathological perforations) or their location (strip, mild root, or apical perforations) [3]. Strip perforation (SP) is an iatrogenic perforation of excessive instrumentation of root canals that occurs during curved roots preparation, especially in the buccal-mesial roots of upper molars, the mesial roots of lower molars, and canals with thin walls [4]. SPs differ from other perforations in their large affected area, irregular perforation site edge, and difficulty in perforation sealing [5]. SPs result in loss of root integrity, damage to adjacent periodontal tissues, and possible contamination of the site, which often leads to inflammation and osseous destruction, and makes the affected area weak and vulnerable to fracture [6].

In perforation management, early diagnosis and an appropriate treatment plan are crucial to the long-term survival of the affected tooth [7]. The diagnosis of perforations is usually achieved by clinical investigations and advanced radiographic examinations [8]. SP is determined clinically by heavy bleeding following an injury. In cases of SP, the bleeding can be detected when a paper point is inserted inside the canal, and if a local anesthetic is not given, unexpected and sudden pain during treatment may also indicate a perforation [6]. Moreover, the use of apex locators is very useful in detecting perforations, as inserting a file into the perforation area will give a zero reading, indicating communication with the periodontal ligament [9]. Radiography is an important part of diagnosing procedural errors like SP during RCT. For example, practitioners can use cone-beam computed tomography, which provides three-dimensional information about the tooth, to evaluate the location of perforation and its diameter [10].

The materials used to treat root perforations should possess several key properties: they should provide adequate sealing, be biocompatible, have stable dimensions, be insoluble and radiopaque, and be easily placed in the root canal [3]. Lately, calcium silicate-based cement has been suggested to treat perforations and support the remaining dental tissues in the injured area [6].

Mineral trioxide aggregate (MTA) is a well-known material originally proposed to repair perforations and has also shown a strengthening effect on the dentinal wall of immature roots. MTA is composed of tricalcium silicate, tricalcium aluminate, tricalcium oxide, and silicate oxide, and has shown many favorable properties, including good sealing, biocompatibility, antibacterial effect, radiopacity, and the ability to set in the presence of blood [11].

Recently, bioceramic putty (EndoSequence, Brasseler, Savannah, GA, USA) has been introduced to the market as a new root-repair material that is available as both a paste in preloaded syringes as well as a moldable putty form. According to the manufacturer, bioceramic putty is composed of calcium silicate, monobasic calcium phosphate, zirconium oxide, tantalum oxide, and filler agents [12]. Bioceramics are biocompatible materials that have antibacterial properties similar to those of MTA [13], and minimal or sometimes no inflammatory response is observed in periodontal tissues after applying these materials [14]. In vitro studies have shown that bioceramic putty has a similar sealing ability compared with MTA [15,16].

Calcium-enriched mixture (CEM) was introduced in 2006 to combine the superior biocompatibility and sealing ability of MTA with the ability to form structures similar to the surrounding dentin, which can stimulate cementogenesis over dentin. CEM consists of a mixture of calcium materials such as calcium oxide, calcium phosphate, calcium carbonate, calcium silicate, calcium sulfate, and calcium hydrate [17].

In light of the controversy surrounding the relationship between SP and the impairment of molars' structure and the ideal material for sealing and supporting SPs, this in vitro study aimed to assess the impairment of molars with SP and the effectiveness of three different calcium silicate cements (MTA, bioceramic putty, and CEM) and gutta-percha in repairing SPs by assessing the fracture resistance of molars with simulated SP in compared to molars without SP.

## Materials and methods

Ethical statement

This experimental in vitro study received ethical approval from the Local Research Ethics Committee of the Faculty of Dentistry, Damascus University (UDDS-522-10102020/SRC-1087), and was self-funded.

Sample selection, preparation, and distribution

This study involved 75 recently extracted, permanent human mandibular first or second molars that were extracted for orthodontic or periodontal reasons. The patients have provided informed consent that their extracted teeth will undergo in vitro study. A periapical X-ray of molars was taken to study their anatomy and root curvature. The exclusion criteria were molars with cracks, carious lesions, open apices, internal or external resorption, double-separated mesial roots, curved canals (>30 degrees), and previous endodontic treatment. The diameter of the mesial root of the molars was measured in the mesial-distal and buccal-lingual directions by an electronic digital caliper (Liaoning MEC Group, Dalian, China), and the mean was determined, where molars whose root diameter exceeded 20% of the mesial-distal or buccal-lingual diameter mean were excluded. Uniform traditional access cavities for all molars were opened using diamond burs and a high-speed handpiece. Apical patency was established with a #10 K-File (Dentsply Sirona, Ballaigues, Switzerland). Then, when the K-File appeared from the apical foramen, the working length was recorded as a reference. The root canals were instrumented with Fanta AF2 (Fanta Dental Materials Co., Ltd., Shanghai, China) to size #25 and taper 0.04, lubricated with ethylenediaminetetraacetic acid (EDTA) 17% gel (Meta Biomed, Cheongju, South Korea), and irrigated with 5.25% sodium hypochlorite solution using a 27G needle (Sybron Endo, Crop, Orange, CA). The solution of EDTA 17% was used as the final irrigation of all canals to remove the smear layer, and finally, all canals were dried with paper points (Gabadent, Guangdong, China). The molars were given numbers, randomized using a randomization site (www.randomization.com), and divided into five main groups as follows: G1 (15 molars): without SP (negative control group); G2 (15 molars): SP filled with gutta-percha (positive control group); G3 (15 molars): SP treated with MTA; G4 (15 molars): SP treated with bioceramic putty; and G5 (15 molars): SP treated with CEM.

**Figure 1:**
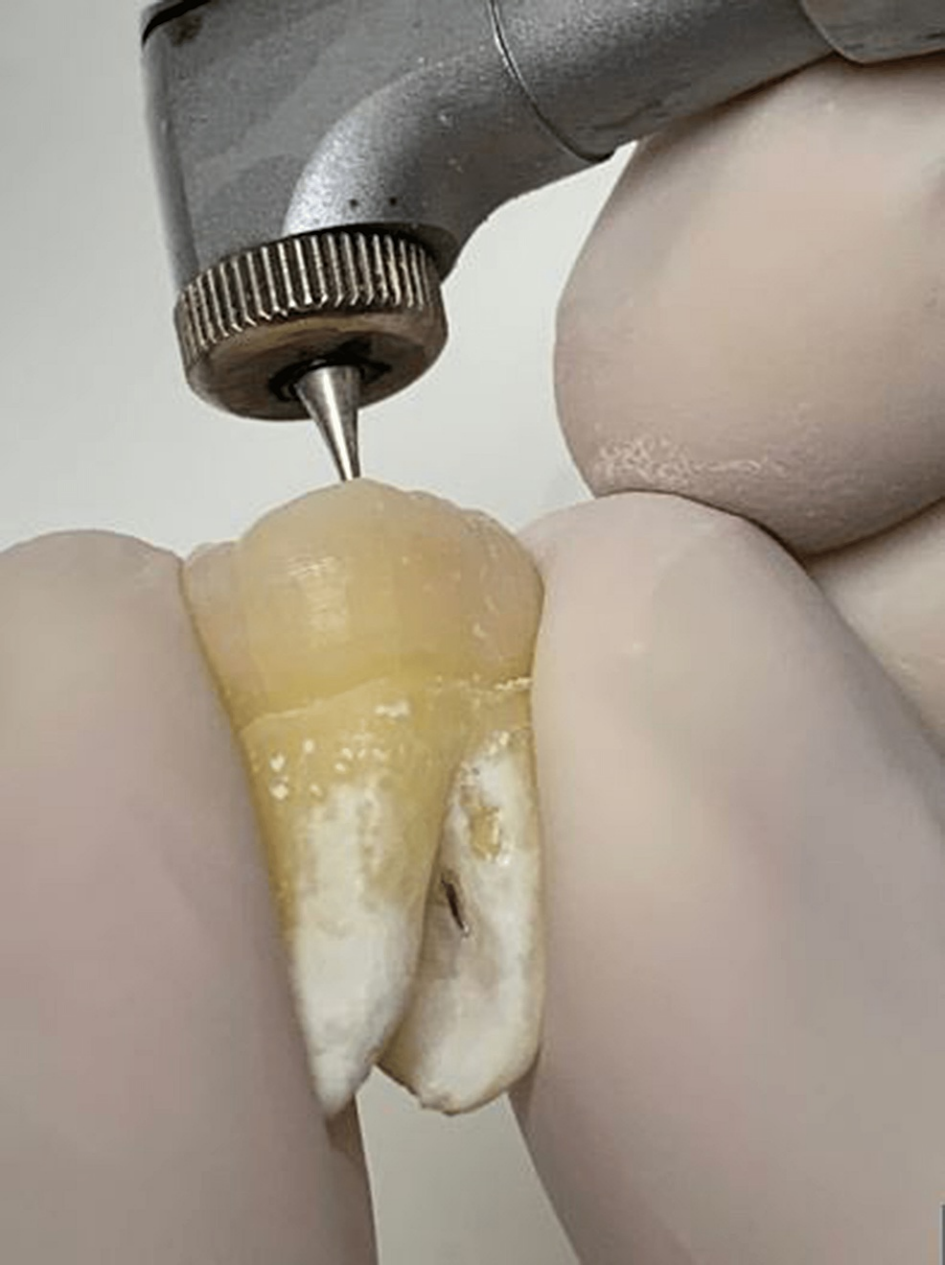
Making stimulated strip perforation using Gates Glidden drill

SPs were made in the molars of G2-G5 at the distal surface of the coronal third of the mesial root, where they were drawn with a felt-tip pen on the outer surface of the root at 3 mm below the furcation area. The distance between the location of the SP and the tip mesiobuccal cusp was measured with a ruler, and then SPs were drilled using Gates Glidden drills (sizes #1-3; Mani, Inc., Tochigi, Japan) (Figure 1).

Then, the roots were embedded in a moist sponge. Warm vertical obturation with gutta-percha (Gabadent, Guangdong, China) was done and AH Plus sealer (Dentsply Sirona Endodontics, Tulsa, OK) was used to fill entire root canals in G1. In contrast, the sandwich technique was used to fill the SP groups (G2-G5) up to their perforation area, where the gutta-percha point was cut level to the perforation area and loaded with AH Plus sealer by placing the tip of a System B device (Kerr Endodontics, Gilbert, AZ) into the canal. Warm vertical obturation was used to gently fill the SPs of G2.

MTA and CEM groups

**Figure 2:**
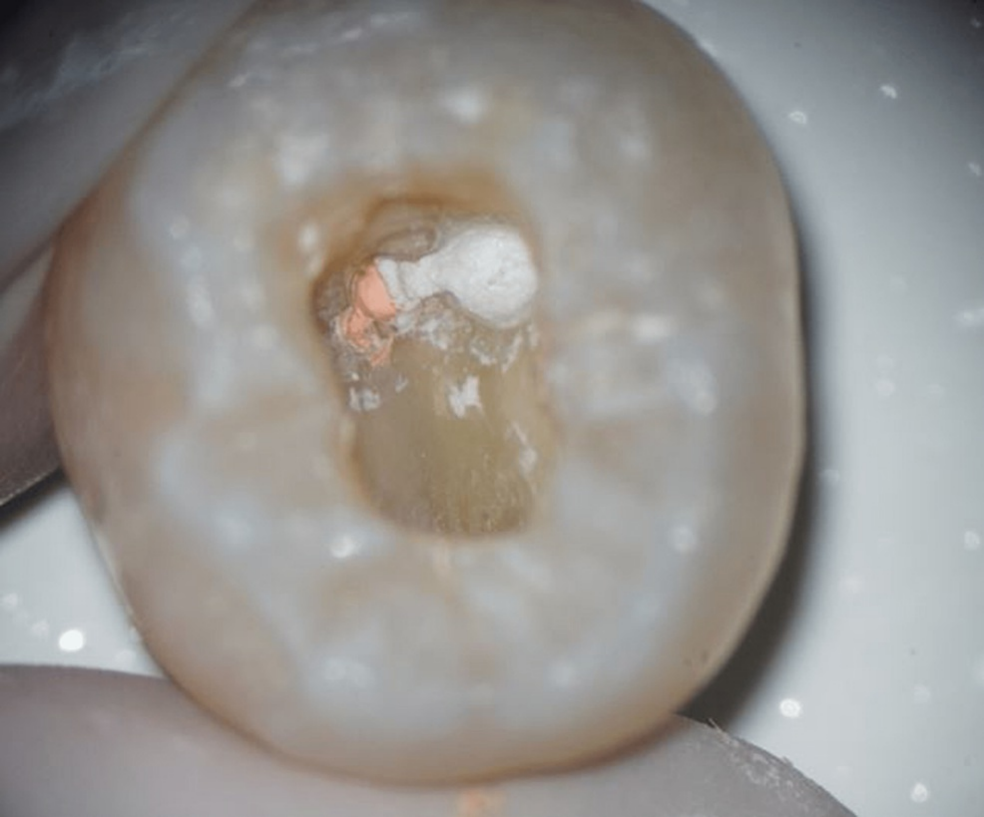
Strip perforation restored with mineral trioxide aggregate

MTA (Produits Dentaires S.A., Vevey, Switzerland) and CEM (Bionique Dent, Tehran, Iran) were mixed according to the manufacturers’ instructions and packed into SP areas using the MAP System® (Produits Dentaires S.A., Vevey, Switzerland) and hand pluggers (Dentsply, Tulsa, OK) (Figure 2).

Bioceramic putty group

Bioceramic putty is a pre-mixed paste that was directly packed into SP areas using the MAP System and hand pluggers.

**Figure 3:**
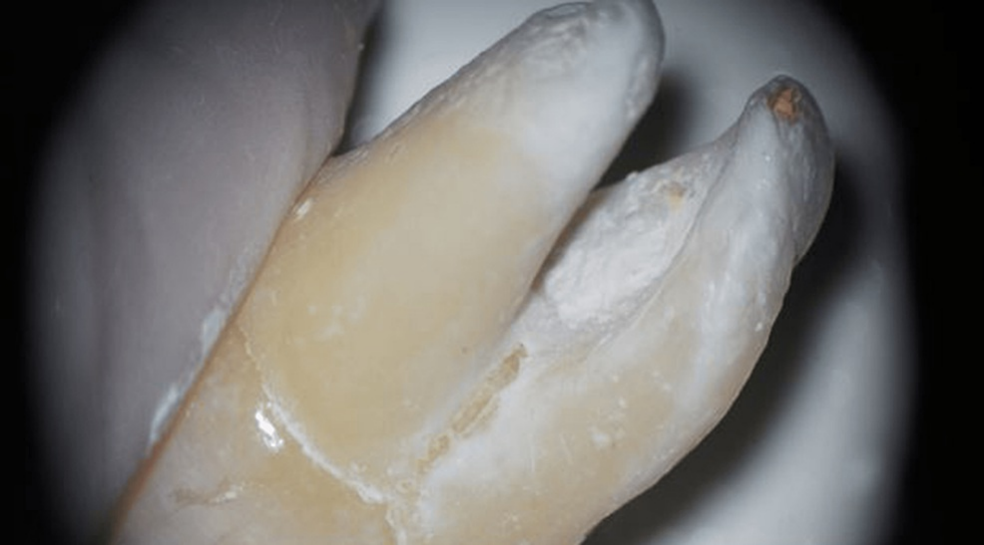
Appearance of strip perforation after trimming

The molars were removed from the sponge, and excess material was trimmed with a scalpel (Figure 3). All molars were restored with composite resin restoration, wrapped in wet gauze, placed in an incubator, and allowed to set for 24 hours at 37°C under 100% humidity before performing fracture resistance tests.

Fracture resistance tests

**Figure 4:**
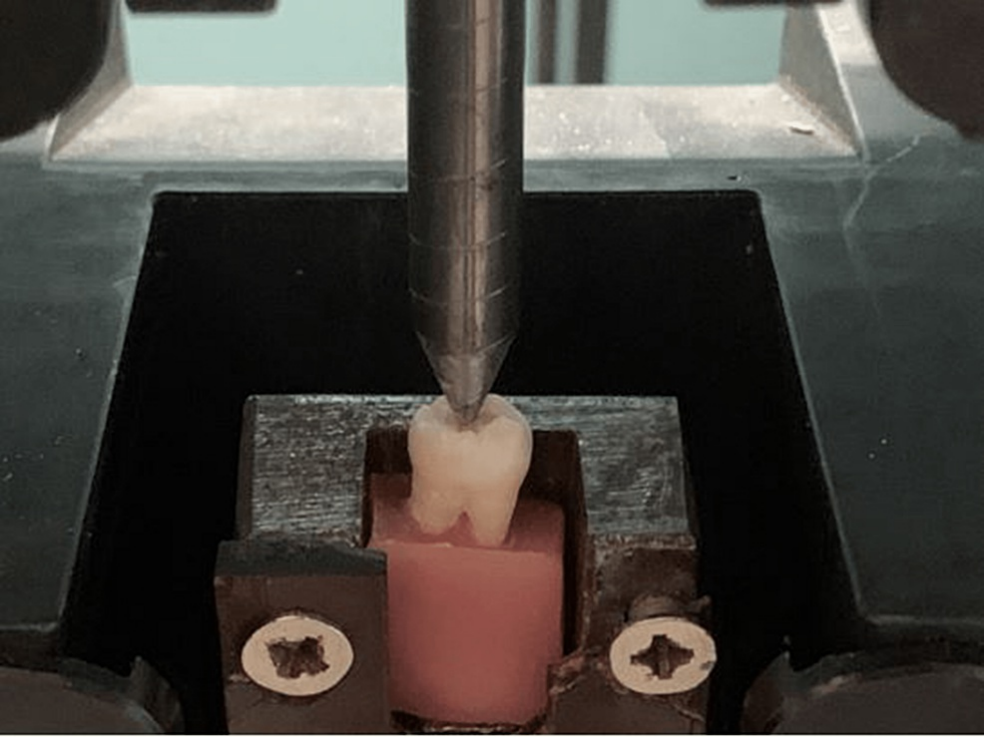
Molars placed in a universal testing machine.

The apical third of all molar roots was covered with a thin layer of polyether impression material; then, the covered area was mounted vertically in (2.5 x 2.5 x 2.5 cm) self-curing acrylic resin blocks. The blocks (including molars) were placed in a universal testing machine (Lloyd LR 30 K, Fareham, England). A compressive vertical loading speed of 0.5 mm/min was applied by spherical tips placed on the center of the occlusal surface of the molar (Figure 4). The force under which the fracture occurred was recorded in newtons.

Statistical analysis

The data were analyzed by a statistician blinded to the material type used in treating SPs using SPSS version 24 software (IBM Corp., Armonk, NY). The distribution of data was evaluated using the Kolmogorov-Smirnov test. As the data were normally distributed, the mean tooth fracture resistance value (in newtons) was compared using a one-way ANOVA test, and pairwise comparisons were made using the Bonferroni test, with the significance level set at p < 0.05.

## Results

Table 1 summarizes the mean, standard deviation, range, and one-way ANOVA test results of the tooth fracture resistance of the molars of the five groups. The one-way ANOVA test showed a significant difference (p = 0.000) between the different groups.

**Table 1:** Descriptive statistics of tooth fracture resistance of the study groups and the p-values of significance testing * One-way ANOVA test. SP: strip perforation; MTA: mineral trioxide aggregate; CEM: calcium-enriched mixture.

Group	Number	Mean ± standard deviation	Range	F-value	P-value*
G1 (without SP)	15	1083.73 ± 99.65	950–1233	76.951	0.000
G2 (SP filled with gutta-percha)	15	656.53 ± 77.16	532–800		
G3 (SP treated with MTA)	15	1025.20 ± 78.46	948–1205		
G4 (SP treated with bioceramic)	15	1034.20 ± 89.48	954–1230		
G5 (SP treated with CEM)	15	794.40 ± 55.82	698–874		

The Bonferroni test was used for pairwise comparisons in Table 2, where it was revealed that G2 (SP filled with gutta-percha) had a lower fracture resistance mean (656.53 N) compared to all other groups (G1 = 1083.73 N, G3 = 1025.20 N, G4 = 1034.20 N, G5 = 794.40 N ; p = 0.000), which was statistically significant. G5 (SP treated with CEM) had a lower fracture resistance mean (794 N) compared to G1, G3, and G4 (p = 0.000 in each pairwise comparison). Moreover, the fracture resistance means of G1 (without SP), G3 (SP treated with MTA), and G4 (SP treated with bioceramic) showed no statistically significant differences in pairwise comparisons (p > 0.05).

**Table 2:** Descriptive statistics of pairwise comparisons between groups and the p-values of significance testing * Bonferroni test. ^ Significant difference.

Group	Compared group	Difference between means	Standard error	P-value*
G5	G4	-239.80	29.74	0.000^
	G3	-230.80	29.74	0.000^
	G2	137.87	29.74	0.000^
	G1	-289.33	29.74	0.000^
G4	G3	9.00	29.74	1.000
	G2	377.67	29.74	0.000^
	G1	-49.53	29.74	1.000
G3	G2	368.67	29.74	0.000^
	G1	-58.53	29.74	0.530
G2	G1	-427.20	29.74	0.000^

## Discussion

The frequency of root perforations occurring during endodontic treatment is 3.85%, and 2.3% of them are SPs [18]. SPs weaken the tooth structure, as they are vulnerable to fracture [6]. Therefore, materials that are suggested to have the reinforcing ability should be preferred for repairing affected areas to prevent fractures [19].

The fracture resistance test can be performed in two ways, either by separating the crown from the root as in some studies [20,21] or by keeping the crowns together with the roots [22,23]. Crowns were preserved in this study for two reasons. Firstly, the ability to reach the perforation site from the access cavity is limited to repair the perforation with the appropriate material, and that is what specialists face while managing these cases. Secondly, defining the force application model so that it simulates the masticatory forces in the mouth, as the force is applied in the crown-apical direction.

The present study aimed to assess the impairment of molars due to SPs and test the reinforcing capability of different calcium silicate-based cements (i.e., MTA, bioceramic putty, and CEM) in the restoration of teeth with simulated SP. SP was simulated in the distal surface of the coronal third of the mesial root by using a Gates Glidden bur to obtain standardization. For the obturation, molars were embedded in a moist sponge to simulate the periapical resistance present in a clinical setting, as well as to facilitate the application of repair materials.

The MAP System, which was used to pack MTA and CEM and seal the affected area tightly, can form good sealed calcium silicate cement plugs compared with other methods [24]. The apical third of all molar roots was covered with a thin layer of polyether impression material to provide a layer simulating the periodontal membrane present in clinical situations, and to protect the extracted molars from the temperature variations during the setting of the acrylic resin.

According to the results of this study, no significant differences were observed among G1 (without SP), G3 (SP treated with MTA), and G4 (SP treated with bioceramic) (1083.73 N, 1025.20 N, and 1034.20 N, respectively), which meant that both MTA and bioceramic can similarly support and restore the correct structure of the tooth as if it was not affected by SP. Many laboratory and clinical studies have confirmed the role of MTA in supporting fragile dental structures and enhancing the survival of weakened teeth within the oral cavity during long follow-up periods, whether in treating immature teeth [25], perforations [21], or various absorptions [26]. Our current study corroborates this finding in SP treatment.

Bioceramic putty represents one of the newer generations of calcium silicate cement (fifth generation) [27]. Through its advanced composition and availability in a pre-mixed paste form, Koç et al. [26] found that it was able to achieve similar success as MTA for sealing root perforations, whereas Abdelmotelb et al. [28] found that it was even better than MTA in sealing root perforations. Similarly, this study found that both MTA and bioceramic putty have a close mean of fracture resistance in restoring molars with SPs.

CEM is a new calcium silicate-based cement [29]. Although CEM has shown some superior results, such as a higher antibacterial effect [30], significantly shorter setting time, easier handling, and no tooth discoloration [31], MTA is superior in microhardness [32] and compressive strength [33]. Moreover, the findings of the current study ensured that MTA had a superior mean of fracture resistance compared with CEM (1025.20 N and 794.40 N, respectively) in terms of SP repairing. A similar result was found in the study of Grayli et al. [34].

The results of this study showed that G2 (gutta-percha-filled SP) had the lowest fracture resistance mean (656.53 N), which ensured the punctual detection of SP during root canal treatment stages because filling the SP with gutta-percha and sealer will expose the tooth to fracture.

The main limitation of this study was the difficulty of finding a larger sample size due to the attempt to standardize the molar measurements included in the research. If there were a greater number of extracted molars, the same study could have been carried out by impacting different SP sizes. In addition, leakage tests could have been performed to assess the ability of the used materials to seal the perforated area.

## Conclusions

Within the above-mentioned limitations of this in vitro study, it can be concluded that SP is a problem that reduces the fracture resistance of endodontically treated molars and it requires additional special materials to repair the affected area. SP restored by MTA and bioceramic putty may provide more strength to the molar structure than restoring with CEM or a gutta-percha/sealer combination. Moreover, MTA and bioceramic putty enhanced the fracture resistance of endodontically treated teeth to levels similar to molars without SP. Consequently, further clinical trials are required to evaluate the clinical performance of MTA, bioceramic putty, and CEM in molars affected by SP.
